# Disturbed blood flow worsens endothelial dysfunction in moderate-severe chronic obstructive pulmonary disease

**DOI:** 10.1038/s41598-017-17249-6

**Published:** 2017-12-05

**Authors:** Otto F. Barak, Suzana Mladinov, Ryan L. Hoiland, Joshua C. Tremblay, Stephen R. Thom, Ming Yang, Tanja Mijacika, Zeljko Dujic

**Affiliations:** 1 Department of Physiology, Faculty of Medicine, University of Novi Sad, Novi Sad, Serbia; 2 Department of Integrative Physiology, University of Split School of Medicine, Split, Croatia; 3 Clinic for Pulmonary Diseases, University Hospital Centre Split, Split, Croatia; 4 Centre for Heart, Lung and Vascular Health, University of British Columbia, Okanagan Campus, Kelowna, British Columbia Canada; 5 Cardiovascular Stress Response Laboratory, School of Kinesiology and Health Studies, Queen’s University, Kingston, Ontario Canada; 6 Department of Emergency Medicine, University of Maryland School of Medicine, Baltimore, Maryland USA

## Abstract

The aims of this study were: (1) to test whether oscillatory shear stress further exacerbates endothelial dysfunction in patients with moderate-severe COPD, and (2) to test whether low flow oxygen administration improves endothelial function and is protective against oscillatory shear stress-induced endothelial dysfunction in patients with moderate-severe COPD. In 17 patients and 10 age-matched non-smoking control subjects we examined brachial artery flow-mediated dilation (FMD) and circulating microparticles before and after 20 minutes of experimentally-induced oscillatory shear stress. COPD patients performed this intervention a second time following a 20-minute wash in period of low flow supplemental oxygen to normalize arterial oxygen saturation. COPD patients had ~six-fold greater baseline retrograde shear rate (P < 0.05) and lower FMD (P < 0.05). The oscillatory shear stress intervention induced significant decreases in brachial artery FMD of all groups (P < 0.05). Oscillatory shear stress elevated circulating markers of endothelial cell apoptosis (CD31+/CD41b− microparticles) in COPD patients, but not age-matched controls. Supplemental oxygen administration abrogated the oscillatory shear stress-induced increase in CD31+/CD41b− microparticles, and improved FMD after accounting for the shear stress stimulus. We have demonstrated that acutely disturbed blood flow with increased retrograde shear stress further deteriorates the already impaired endothelial function with attendant endothelial apoptosis in patients with moderate-severe COPD.

## Introduction

Chronic obstructive pulmonary disease (COPD) is a chronic inflammatory disease of the lungs that exerts complications systemically, highlighted by an increased risk of cardiovascular disease. Accordingly, the main cause of death among these patients is cardiovascular events^1–3^, yet the exact mechanisms underpinning this increased risk of cardiovascular events in COPD are not well understood with systemic endothelial dysfunction proposed as a contributory factor^4–7^. Patients with COPD present with elevated levels of circulating endothelial-derived microparticles (EMPs) and impaired flow-mediated dilation (FMD), indicating a pro-atherogenic endothelial cell phenotype; however, whether the endothelium of patients with COPD remains sensitive to oscillatory shear stress-induced dysfunction remains unknown.

The magnitude and direction of shear stress exerted upon the endothelium mediates gene expression; laminar, pulsatile shear stress induces a quiescent endothelial cell phenotype that is anti-inflammatory, vasodilatory, and antithrombotic, whereas oscillatory, low time-averaged mean shear stress is pro-atherogenic^8^. Studies in young, healthy humans indicate that 20–30 minutes of experimentally-induced oscillatory shear stress increases EMP markers of endothelial cell activation and apoptosis, and decreases FMD^9,10^. The influence of disturbed hemodynamics on endothelial function has never been assessed in patients with COPD. Further, acute hypoxemia increases circulating MPs, while superimposing oscillatory shear stress impairs FMD in young, healthy humans^11^. Hypoxemia is a characteristic of moderate-severe COPD; however, the potential link between chronic hypoxemia and EMPs in COPD has yet to be investigated.

The primary aims of this study were two-fold: 1) to test whether oscillatory shear stress further exacerbates endothelial dysfunction in patients with COPD compared to age-matched controls, and 2) to test whether low flow oxygen administration, to alleviate hypoxemia, improves endothelial function and is protective against oscillatory shear stress-induced endothelial dysfunction in patients with COPD.

## Results

Characteristics of the study population are given in Table 1.

**Table 1:** Characteristics of the study population.

	Control subjects	COPD
	(n = 10)	(n = 17)
Age (years), mean (SD)	65.3 (7.3)	69.0 (8.1)
Sex (male), No. (%)	7 (70.0)	11 (64.7)
Height (cm), mean (SD)	174.5 (9.0)	170.6 (8.0)
Weight (kg), mean (SD)	89.1 (13.3)	76.5 (16.8)
Body mass index (kg/m^2^), mean (SD)	29.3 (4.0)	26.2 (5.2)
Cigarette smoking status		
Current, No. (%)	0 (0.0)	4 (23.5)
Former, No. (%)	4 (40.0)	11 (64.7)
Non-smoker, No. (%)	6 (60.0)	2 (11.8)
Smoking pack-years, mean (SD)	28.8 (22.5)	54.3 (62.8)
Hypertension, No. (%)	4 (40.0)	11 (64.7)
Diabetes mellitus, No. (%)	1 (10.0)	3 (17.6)
Coronary artery disease, No. (%)	0 (0.0)	1 (5.9)
Peripheral artery disease, No. (%)	0 (0.0)	2 (11.8)
Medication use		
β-blockers, No. (%)	1 (10.0)	4 (23.5)
Calcium channel blockers, No. (%)	0 (0.0)	3 (17.6)
ACE inhibitors or angiotensin antagonists, No. (%)	0 (10.0)	8 (47.1)
Diuretics, No. (%)	1 (10.0)	12 (70.6)
Statins, No. (%)	0 (0.0)	1 (5.9)
Acetylsalicylic acid, No. (%)	0 (0.0)	2 (11.8)
Inhaled corticosteroids, No. (%)	0 (0.0)	14 (82.4)
Systemic corticosteroids, No. (%)	0 (0.0)	1 (5.9)
Short-acting β-agonists, No. (%)	0 (0.0)	4 (23.5)
Long-acting β-agonists, No. (%)	0 (0.0)	17 (100.0)
Short-acting anticholinergics, No. (%)	0 (0.0)	3 (17.6)
Long-acting anticholinergics, No. (%)	0 (0.0)	13 (76.5)
Theophylline, No. (%)	0 (0.0)	7 (41.2)
Roflumilast, No. (%)	0 (0.0)	2 (11.8)
Methyldigoxine, No. (%)	0 (0.0)	1 (5.9)
Ivabradine, No. (%)	0 (0.0)	1 (5.9)
Systolic blood pressure (mm Hg), mean (SD)	133.0 (18.7)	133.2 (21.8)
Diastolic blood pressure (mm Hg), mean (SD)	84.0 (7.4)	78.8 (12.6)
FEV_1_ (% of predicted), mean (SD)	107.3 (22.5)	31.8 (11.0)
FVC (% of predicted), mean (SD)	110.8 (24.6)	52.3 (10.8)
FEV_1_/FVC ratio (%), mean (SD)	77.7 (6.0)	45.9 (10.1)
FEF_25–75_ (% of predicted), mean (SD)	88.9 (23.4)	10.9 (4.3)
SaO_2_ (%), mean (SD)	96.4 (1.0)	88.2 (3.7)
PaO_2_ (kPa), mean (SD)		7.2 (0.9)
PaCO_2_ (kPa), mean (SD)		6.3 (1.0)
pH, mean (SD)		7.42 (0.03)
HCO_3_ (mmol/L), mean (SD)		30.5 (4.9)
Home oxygen therapy, No. (%)		14 (82.4)
O_2_ washout period (h), mean (SD)		6.5 (5.8)
Severity of airflow limitation (GOLD)		
Stage 1, No. (%)		0 (0.0)
Stage 2, No. (%)		1 (5.9)
Stage 3, No. (%)		6 (35.3)
Stage 4, No. (%)		10 (58.8)
Dyspnea grade (mMRC), mean (SD)		3.4 (0.6)
Acute exacerbations ≥2 per year, No. (%)		12 (70.6)
Combined COPD assesment		
Group A, No. (%)		0 (0.0)
Group B, No. (%)		1 (5.9)
Group C, No. (%)		0 (0.0)
Group D, No. (%)		16 (94.1)

COPD = chronic obstructive pulmonary disease; ACE = angiotensin-converting enzyme; FEV_1_ = forced expiratory volume in the first second; FVC = forced vital capacity; FEF_25–75_ = forced expiratory flow between 25% and 75% of FVC; SaO_2_ = arterial blood oxygen saturation; PaO_2_ = arterial blood oxygen tension; PaCO_2_ = arterial blood carbon dioxide tension; GOLD = Global Initiative for Chronic Obstructive Lung Disease; mMRC = modified Medical Research Council.

Baseline systolic, diastolic and mean blood pressures were not different between the two interventions, or between the two groups (Table 2).

**Table 2:** Blood pressure and oxygen saturation of subjects before and after the intervention Values are mean ± SD.

	COPD patients - room air		COPD patients - 100% O_2_		Control subjects	
	Pre intervention	Post intervention	Pre intervention	Post intervention	Pre intervention	Post intervention
SBP (mmHg)	130.9 ± 18.3	131.2 ± 17.5	130.0 ± 17.8	133.2 ± 21.1	132.5 ± 18.7	133.5 ± 18.3
DBP (mmHg)	78.2 ± 11.9	77.6 ± 9.7	77.9 ± 9.8	79.2 ± 13.3	83.0 ± 7.1	84.0 ± 7.0
MAP (mmHg)	95.8 ± 13.5	95.5 ± 11.5	95.3 ± 11.9	97.2 ± 15.4	99.5 ± 10.8	100.5 ± 10.4
SaO_2_ (%)	88.1 ± 3.8	87.9 ± 3.7	97.1 ± 1.2^*^	97.1 ± 0.9^*^	96.8 ± 1.0^†^	96.9 ± 0.9^†^

SBP, systolic blood pressure; DBP, diastolic blood pressure; MAP, mean arterial pressure, SaO2, oxygen saturation.

*Indicates significant difference between room air and 100% O2 conditions (P < 0.05).

^†^Indicates significant difference between COPD patients and control subjects at room air condition (pre and post intervention) (P < 0.05).

**Figure 1:**
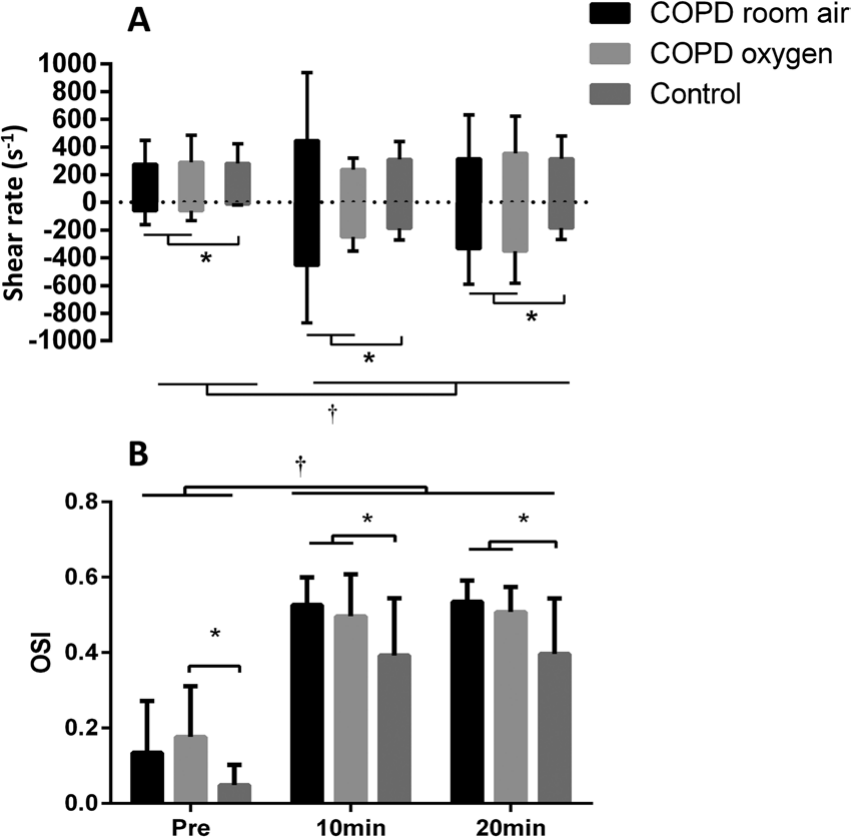
Shear rate patterns (panel A) and oscillatory shear index (OSI) (panel B) before and during (10^th^ and 20^th^ minute) the intervention of cuff inflation to 75 mmHg on the experimental arm. Data of anterograde and retrograde shear rates (panel A) and oscillatory shear index (panel B) are presented for COPD patients exposed to room air (black bars) and 100% oxygen (light grey bars) and control subjects breathing room air (dark grey bars). Error bars represent SD. *Indicates significant difference between groups ( P< 0.05). ^†^Indicates significant difference from baseline (P < 0.05).

**Figure 2:**
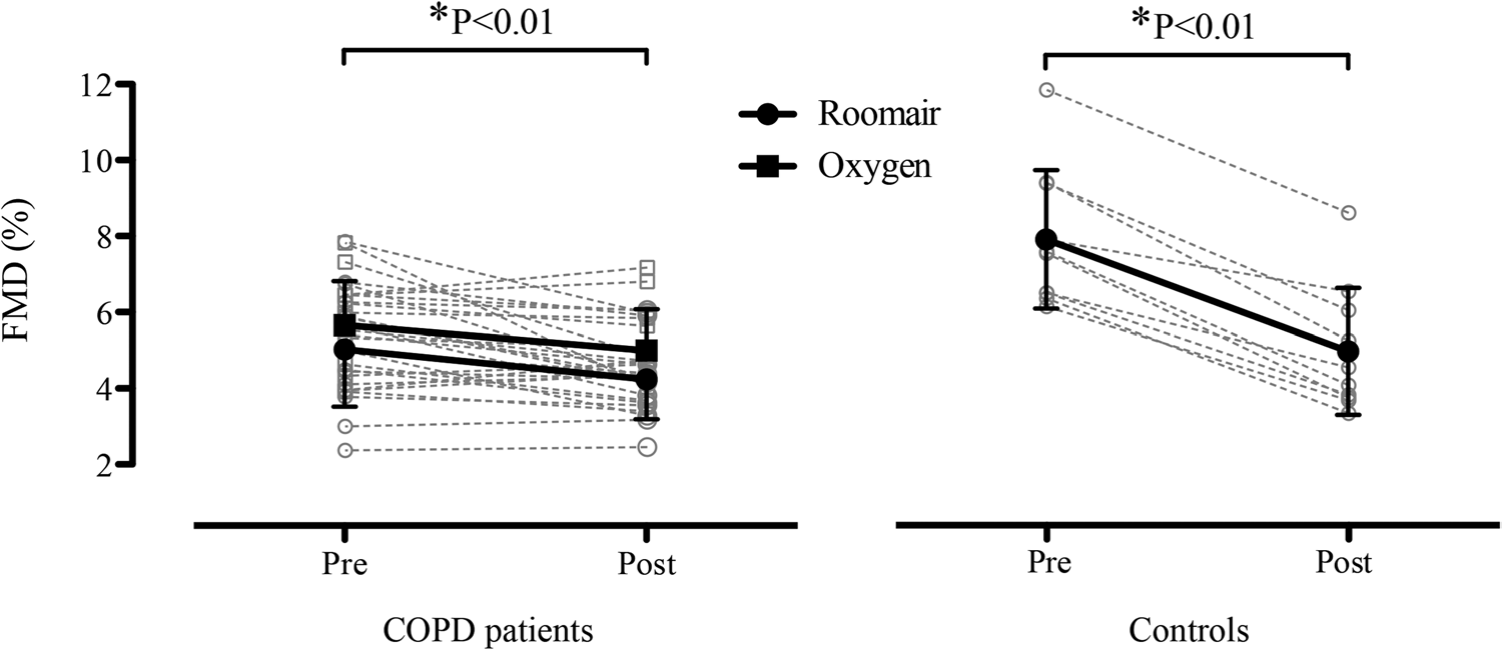
COPD and control subject FMD of the experimental arm following elevated retrograde shear. There was a main effect of retrograde shear ( P< 0.05) during the COPD trials in room air and during oxygen breathing. In the control subjects, FMD was similarly reduced (P < 0.05) following elevated retrograde shear during room air breathing.

Pre-intervention, 10-minute, and 20-minute shear rate patterns were significantly different between COPD and controls, with COPD patients displaying greater retrograde shear rate both before and during O_2_ reversal ( P< 0.05) (Fig. 1). In COPD and controls twenty-minute cuff inflation to 75 mmHg increased retrograde shear rate (P < 0.05) but did not alter anterograde shear rate (P > 0.05) (Fig. 1). Differences in the oscillatory shear index (OSI; |retrograde shear rate|/[|antegrade shear rate| + |retrograde shear rate|]) between groups and following cuff inflation mirrored the changes in retrograde shear rate. The only difference was that at baseline, OSI was only lower in controls compared to the COPD patients during O_2_ normalization – otherwise OSI was always lower in controls (P < 0.05), with OSI increasing in all groups following cuff inflation (P < 0.01). At rest, FMD was 36% lower in the COPD patients than in controls (5.03 ± 1.51 vs. 7.88 ± 1.81%; P < 0.01). Mean arterial pressure (MAP) as a covariate had no effects on baseline FMD (F = 0.048, P > 0.05). The covariates ratio of forced expiratory volume in the first second and forced vital capacity (FEV_1_/FVC ratio) and oxygen saturation (SaO_2_) had significant effects on baseline FMD (F = 6.588, P < 0.05, partial η^2^ = 0.223 and F = 22.131, P < 0.01, partial η^2^ = 0.490, respectively). The retrograde intervention induced significant decreases in brachial artery FMD (absolute terms) and FMD% (relative terms) in the experimental arm of all groups, including COPD following O_2_ normalization ( P< 0.05) (Fig. 2; Table 3). This effect of the retrograde occurred after inclusion of baseline diameter and SRAUC as covariates. None of the considered covariates had significant effects on the FMD response to oscillatory shear stress: MAP (F = 0.510, P > 0.05), FEV_1_/FVC ratio (F = 0.014, P > 0.05) and SaO_2_ (F = 0.639, P > 0.05). MAP and FEV_1_/FVC ratio had no significant effects on the FMD response to low flow oxygen supplementation in COPD patients (F = 2.531, P > 0.05 and F = 3.539, P > 0.05, respectively), but SaO_2_ did have a significant effect (F = 5.006, P < 0.05, partial η^2^ = 0.152). In COPD patients a 15% decrease in FMD% due to oscillatory shear stimulus during breathing room air and 12% during breathing O_2_ had moderate effect size (0.60 and 0.59, respectively). The decrease in FMD% of about 37% in controls had an effect size of 1.69. There was a moderate correlation between baseline FMD and retrograde shear in COPD patients (r = 0.573, P = 0.07) but no correlation was observed in controls (r = 0.009, P = 0.98). Further, after inclusion of baseline diameter and SRAUC as covariates, FMD was greater during O_2_ normalization than room air breathing in patients with COPD (5.5% *versus* 4.2%; P < 0.05).

**Table 3:** Brachial artery flow mediated dilation pre and post intervention on the experimental arm.

	COPD patients – Room air		COPD patients - 100% oxygen		Control subjects	
	Pre intervention	Post intervention	Pre intervention	Post intervention	Pre intervention	Post intervention
Baseline diameter (mm)	4.69 ± 0.87	4.81 ± 0.81	4.74 ± 0.87	4.71 ± 0.91	6.49 ± 0.77^†^	6.45 ± 0.76^†^
Peak diameter (mm)	4.92 ± 0.92	5.01 ± 0.85	5.01 ± 0.91	4.94 ± 0.93	7.01 ± 0.86^†^	6.77 ± 0.81^†^
Time to peak (s)	107.3 ± 31.6	93.9 ± 29.0	92.9 ± 22.5	91.6 ± 24.9	94.6 ± 23.2	89.7 ± 24.4
FMD (mm)	0.24 ± 0.09	0.21 ± 0.07^*^	0.27 ± 0.07	0.23 ± 0.05^*^	0.52 ± 0.15^†^	0.32 ± 0.12^*†^
SRAUC (s^−1^)	25876 ± 9985	17507 ± 6005^*^	18252 ± 6732^††^	14198 ± 3976^*††^	26124 ± 5289	21811 ± 5289^*^

*Indicates significant difference between pre and post intervention measures (P < 0.05).

^†^Indicates significant difference between COPD and control subjects at room air conditions (pre and post intervention) ( P< 0.05).

^††^Indicates significant difference between COPD patients – Room air and COPD patients – 100% oxygen (P < 0.05).

**Figure 3:**
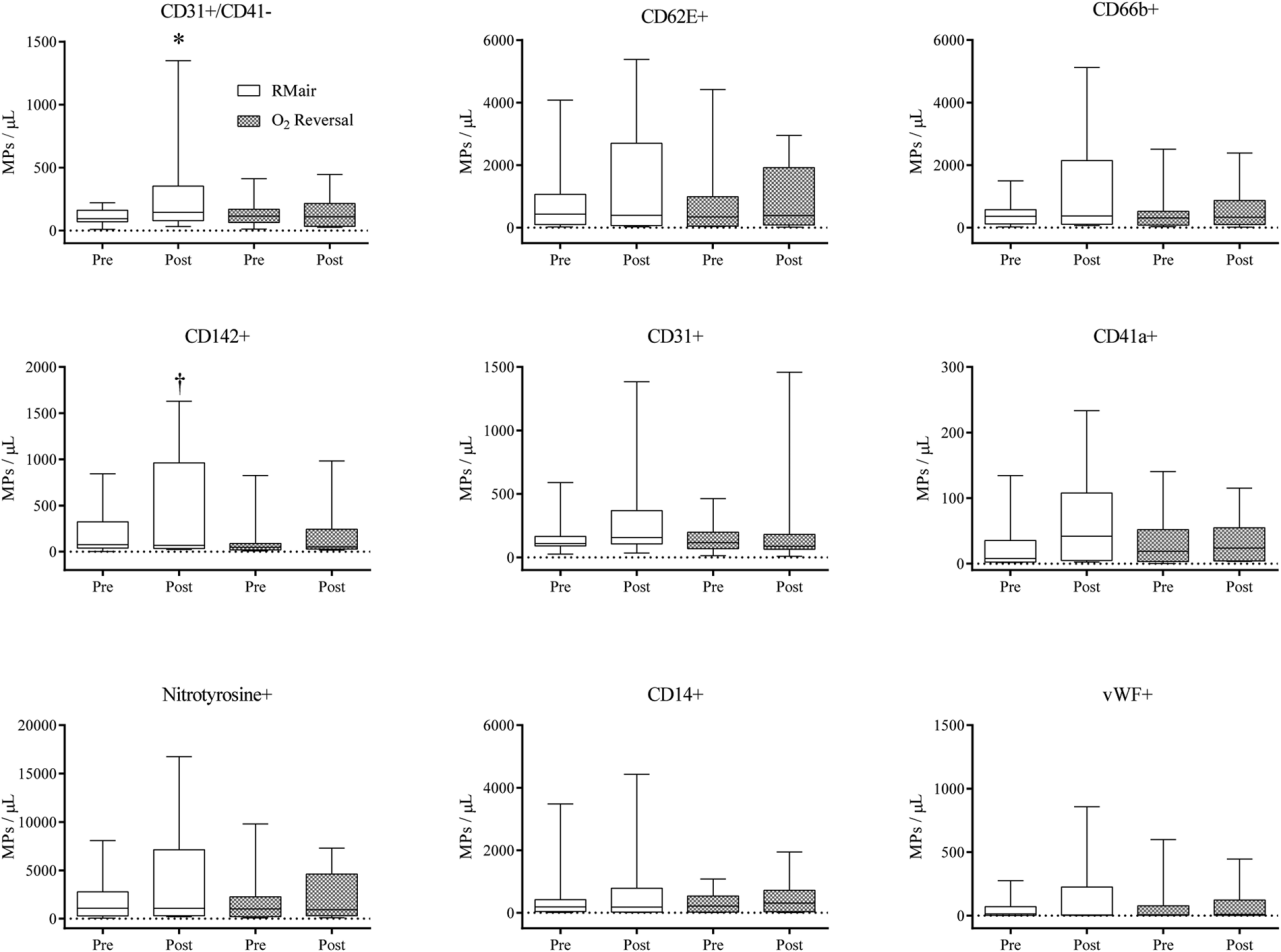
Baseline and post intervention MP levels in COPD patients breathing room air and 100% oxygen. Room air values are represented by white box plots, and the O_2_ reversal data by grey patterned box plots. *Indicates significant difference from room air pre-retrograde. ^†^Indicates significant difference from O_2_ reversal pre-retrograde.

Plasma MP changes for the experimental arm in COPD patients are shown in Fig. 3. There was a significant interaction (P < 0.05) for CD31+/41b−, CD66b+ and CD142+ while no significant effect was detected for the other MPs. Post-hoc tests revealed that CD31+/41b− was elevated following retrograde shear stress (P < 0.05) during room air breathing, but this increase was absent following O_2_ normalization. There was no correlation between the change in CD31+/41− EMPs and FMD in the COPD patients – for both room air and O_2_ breathing. There were no changes in MP levels in the control arm of COPD patients. In control subjects there was no impact of retrograde shear stress (P > 0.05) or experimental vs. control arm (P > 0.05) on any MP sub-type levels.

## Discussion

The main novel findings of this study are: (1) in patients with moderate-severe COPD, acute increases in retrograde shear stress and/or reductions in mean shear stress further deteriorate the already impaired endothelial function with attendant endothelial apoptosis, and (2) that supplemental O_2_ abrogates the oscillatory shear stress-induced increase in CD31+/41b− and improves endothelium-dependent vasodilation for a given stimulus. However, the mechanisms underlying these changes seem to be different in COPD patients compared to age-matched controls. In patients with moderate-severe COPD, the oscillatory shear stress induces increases in circulating markers associated with endothelial apoptosis that is paralleled by a modest attenuation of endothelial function (measured via FMD). Moreover, at rest the lower FMD of COPD patients compared to controls is accompanied by a correspondingly elevated baseline retrograde shear rate. This indicates that disturbed blood flow may contribute to increased cardiovascular risk in this population by exacerbating endothelial dysfunction^12^. In apparently healthy older adults, a large attenuation in FMD following oscillatory shear stress is not coupled with an increase in CD31+/41b− MP, suggesting mechanisms unrelated to EMP release or clearance. Collectively, these data for the first time provide *in vivo* evidence that links the detrimental role of pro-atherogenic events (i.e., oscillatory shear stress) with endothelial dysfunction in moderate-severe COPD patients.

Endothelial dysfunction is regarded as an early manifestation of atherogenic events and vascular injury^13^. Given its presence in COPD patients, the main question of this study was if disturbed blood flow, which is a pro-atherogenic stimulus, would further deteriorate endothelial function^14^. We have shown that acute periods of oscillatory shear stress lead to further endothelial damage, decreasing brachial FMD while elevating CD31+/CD41b− MP, which is indicative of endothelial apoptosis. Although all measures have been undertaken to limit bias based upon image interpretation/discrimination (e.g., coding files prior to analysis) the delta of less than 1% in FMD reduction in COPD patients following the acute intervention of retrograde shear stress manipulations requires discussion of data with caution. However, this small reduction in FMD may be quite important, as it occurred in spite of previous data demonstrating that individuals with *a priori* low FMD do not undergo a further reduction in FMD following a retrograde shear stress stimulus^15^. This may reflect an augmented susceptibility to endothelial damage/dysfunction in COPD, suggesting that despite COPD patients having dysfunctional endothelium are still susceptible to further deterioration. Interestingly, an increase in circulating CD31+/CD41b− expressing MP and decrease in FMD manifests during COPD exacerbations^7,16^, suggesting a potential mechanism linking the increased risk of cardiovascular event following exacerbation^1^. Further, although a notably small change, it may also carry a significant weight with regards to cardiovascular risk^17^.

Concurrent to the *a priori* reduced endothelial function, COPD patients in our study had higher resting retrograde shear rate as compared to healthy controls. To our knowledge this is the first study to examine shear patterns and retrograde shear rate in moderate-severe COPD patients. A laminar, pulsatile shear pattern in conduit arteries mainly presents as blood flow in the anterograde direction but increased retrograde flow patterns may manifest from increased vascular tone in the downstream resistance vessel^18^. This is commonplace in advanced age, obesity, and hypertension^15,19,20^. We have now demonstrated that retrograde shear rate may contribute to endothelial dysfunction in patients with COPD.

We have observed a decrease in FMD% in apparently healthy aged controls after generating retrograde shear stress that is not in accordance to previous findings of Schreuder *et al*.^15^. One possible explanation could be the presence of a threshold shear rate stimulus, which has dose-dependent effects on reducing FMD%^9^. This has implications considering the protocol implemented by Schreuder *et al*. did not use cuff inflation above 60 mmHg, nor did they employ a second cuff on the upper arm^15^. In a control experiment, Jenkins *et al*. found that the proximal 40 mmHg did not produce changes in MP, but there were slight increases in retrograde shear rate^10^, which may have contributed to the reduction in FMD observed in the current study. In control subjects, the 20-minute retrograde shear stress intervention did not cause any significant endothelial damage (no increase in MP) yet there was a large decrease in the subsequent FMD. Therefore, while retrograde shear stress impairs the endothelium in COPD patients at the cellular and functional level, other mechanisms downstream of retrograde shear stress manipulation must impair FMD in healthy controls, such as a decrease in endothelial NO synthase expression^21^ and/or elevated oxidative stress^22^.

The retrograde shear stress intervention induced elevations in markers of endothelial cell apoptosis, CD31+/41b− MP. Unlike the Jenkins study, where complete distal occlusion (220 mmHg) was applied to increase retrograde shear stress, we used only 75 mmHg^10^, which lead to endothelial cell apoptosis (e.g., increased CD31+/41b−) but not activation (e.g., unchanged CD62E+).

The specific cellular mechanisms by which systemic inflammation plays an important role in the pathogenesis of cardiovascular disease in COPD are complex. The atherosclerotic process starts with the injury of the vascular endothelium that is even more exposed to damage in the presence of increased retrograde shear stress (increased markers of endothelial apoptosis following acute flow disturbance). Oscillatory shear stress with elevations in retrograde shear showed increased expression of adhesion molecules and elevated endothelin-1 expression, which would allow circulating leukocytes to adhere to the damaged endothelial surface^21^. However, in this study we did not observe shear stress-induced elevations of MPs bearing markers from leukocytes, neutrophils, or platelet-endothelial adhesion molecules in COPD patients.

Administration of low-flow supplemental oxygen appeared to improve FMD after covarying for changes in SRAUC in COPD patients. As hypoxia increases carotid body output^23^ and sympathetic outflow to vascular beds^24^, we reasoned that normalization of oxygen saturation would reduce sympathetic outflow^24^ and alleviate the FMD impairments associated with hypoxia-mediated sympathoexcitation^25^. A previous investigation demonstrated that in healthy individuals sympathetic outflow remains elevated for ≥20-minutes following 20 minutes of moderate (80% SaO_2_) hypoxia^26^, however, this elevation in sympathetic outflow is mitigated with hyperoxia^24^. Despite this rationale for low-flow oxygen therapy, the authors are fully aware that correcting hypoxemia with 20-minutes of supplemental oxygen is not likely to fully reverse the vascular effects of chronic (i.e., years) hypoxemia. However, short-term application (20 minutes) of nasal oxygen has previously been demonstrated to elicit a modest decrease in muscle sympathetic nerve activity^27^. We found no related influence on the retrograde shear pattern and we observed that FMD at rest was unchanged despite a smaller stimulus, which may suggest some improvement in endothelial function. Another consideration is that endothelial dysfunction is initiated early in COPD when airflow obstruction is mild to moderate but arterial hypoxemia is absent, indicating that hypoxia may not be directly related to the observed reductions in FMD in moderate-severe COPD patients, however, our investigation is the first to explore this role in moderate-severe patients. In agreement with the present study, Dinh-Xuan *et al*. demonstrated correlation, albeit not causation, of impaired endothelium-dependent dilation and reduced PaO_2_ values *in vitro*, suggesting that chronic hypoxemia contributes to the impairment in endothelial function in COPD patients^28^. Of note, oxidative stress has been implicated as a contributor to endothelial dysfunction in COPD patients^29^; however, 20-minutes of oxygen therapy does not change plasma levels of oxidative and inflammatory markers^30^. Therefore, it is unlikely the changes observed in the current study are due to altered redox balance.

It is intriguing that the oscillatory shear stress-induced elevation in CD31+/41b− was abolished in the oxygen normalization trial (Fig. 3). As the oscillatory shear stress stimulus was the same in the room air and oxygen normalization trial, a localized effect of oxygen, independent of oscillatory shear stress must have elicited a protective effect. As hypoxia and oscillatory shear stress have been implicated in the rise in circulating MP, it appears as though retrograde shear stress may require a secondary insult to disrupt endothelial cell quiescence. Accordingly, arterial segments chronically exposed to athero-prone shear patterns require further provocation, such as hyperlipidemia, to develop atherosclerosis^8^. Thus, in patients with COPD, we propose that hypoxemia contributes to endothelial dysfunction by promoting endothelial dysfunction that can be amplified by oscillatory shear stress. After normalizing FMD for baseline diameter and SRAUC, endothelial function was significantly improved during O_2_ administration in patients with COPD. The improvement in FMD may implicate a role for hypoxia in restricting endothelial sensitivity to increases in shear stress, supporting previous *in vitro* findings^28^. Thus, it appears as though both mechanical (oscillatory shear stress) and chemical (hypoxia) insults may contribute to and worsen endothelial dysfunction in patients with COPD.

Although 20-minute cuff inflation to 75 mmHg is not likely to induce ischemia-perfusion injuries we wanted to be sure there was no blood contamination from the distal ischemic region prior to sampling. This question was addressed through measuring lactate concentration in the experimental arm at the beginning and at the end of the cuff inflation and we found no significant increase. We did not measure FMD on the contralateral arm, which served as an internal control, since in the work of Thijssen *et al*. they did not observe changes in the pattern of SR or in FMD%^9,22,31^. After inclusion of baseline diameter and SRAUC as covariates, FMD did not show a statistically significant reduction following the retrograde shear stress intervention in patients with COPD. While FMD% was decreased following the intervention, the accompanying reduction in SRAUC, the stimulus for FMD, suggests that endothelial sensitivity to shear stress was not impaired by the oscillatory shear stress intervention. However, a full stimulus-response relationship between shear rate and FMD was not acquired; whether for a given shear stress stimulus FMD is impaired following disturbed blood flow in patients with COPD remains unknown.

Methodological considerations. Clinically, the aim of oxygen therapy is to increase PaO_2_ to >60 mmHg, and thus render a patient’s SaO_2_ ~90% - this has been demonstrated to reduce mortality in this patient population^32,33^. This is in accordance with the British Thoracic Society Guidelines, which indicate 88–92% saturation as the target of intervention in Type II respiratory failure. In the present study, we used a supervised short-term intervention of low flow (e.g., 3 L/min) oxygen to achieve a SaO_2_ of 98% in our COPD patients. This allowed for comparisons with controls who posses a SaO_2_ of 98% at rest. As controls were already normoxic, no intervention was necessary to “restore” an already intact peripheral oxyhemoglobin saturation. Indeed, superimposing oxygen supplementation onto a healthy SaO_2_ (~98%) can lead to multiple physiological consequences such as altered sympathetics

^34^, oxidative stress^35^, nitric oxide bioavailability^36^, and peripheral vascular function^37^. This would have rendered a comparison between O_2_ supplemented COPD patients and O_2_ supplemented controls highly confounded, which rationalized the exclusion of such a trial from the present study.

In COPD endothelial dysfunction presents alongside elevated levels of resting retrograde shear rate. Acutely disturbed blood flow characterized by an oscillatory shear stress pattern deteriorates endothelial function in both patients and controls, however in moderate-severe COPD patients such dysfunction is compounded with attendant increases in circulating MPs indicative of endothelial cell apoptosis, and is of greater potential consequence given the already impaired vasculature of this population. Administration of oxygen abolishes the oscillatory shear stress-induced increase in circulating MP, and appears to improve FMD for a given stimulus. Examining whether eliminating retrograde shear and augmenting mean shear (i.e. via limb heating) improves endothelial function in patients with COPD may provide additional support that disturbed blood flow contributes to endothelial dysfunction in this population.

## Methods

### Study population

The study population consisted of 17 patients (69 ± 8 years) with stable COPD (no exacerbations in the previous six weeks), and 10 age-matched non-smoking control subjects without airflow obstruction (65 ± 7 years). The 14 patients who were on home oxygen therapy used stationary oxygen concentrators that deliver O_2_ in a concentration higher than 90% with a flow 1–2 L/min. Inclusion criteria for patients were defined as moderate-to-severe airflow obstruction on post-bronchodilator spirometry and arterial oxyhemoglobin saturation (SaO_2_) below 90% at rest. COPD was diagnosed and classified according to the Global Initiative for Obstructive Lung Disease (GOLD). All procedures were performed in accordance with the Declaration of Helsinki and the study was approved by the Ethics Committee of the University of Split School of Medicine. Written, informed consent was obtained from all participants. The study was registered at ClinicalTrials.gov under NCT03004352 identifier on December 22^nd^, 2016. The datasets generated during and/or analyzed during the current study are available from the corresponding author on reasonable request.

### Study design

Prior to their arrival at the laboratory, participants were instructed to abstain from exercise, alcohol and caffeine for a minimum of 12 hours and were fasted for 4 hours prior to the testing session. Current smokers had their last cigarette ≥8 hours before the measurements. Patients and controls remained on their prescribed medication during the study. All experimental measures took place in a quiet, temperature-controlled (20–22 °C) room in the Laboratory for Integrative Physiology, University of Split School of Medicine. Venous catheters were placed in the superficial antecubital vein of the right (experimental) and left (control) arms while participants were rested supine. Following 20-minutes rest, blood samples were taken from both arms, and measures of brachial artery hemodynamics and FMD were acquired. Immediately after this assessment, an occlusion model adopted by Jenkins *et al*.^10^ was modified to induce retrograde flow within the experimental arm lasting for 20 minutes. Briefly, a pneumatic cuff placed immediately distal to the epicondyles was inflated to 75 mmHg to provoke low time-averaged shear stress with a high retrograde component. A second pneumatic cuff was placed close to the axilla and inflated to 40 mmHg to facilitate trapping of circulating MPs. The contralateral (control) arm was cuff-free during this intervention. Measures of brachial artery shear rate were taken 10 and 20 minutes into the intervention and blood samples were taken from the experimental and control arms just before cuff deflation. Following a five-minute recovery after cuff release another FMD measurement was made.

COPD patients underwent the testing as outlined at rest breathing room air and following 20-minutes of low flow supplemental oxygen (100%) administered through a nasal cannula in order to normalize arterial oxygen saturation (≥98% SpO_2_). The normalized arterial oxygen saturation was maintained throughout the second set of tests. Control subjects were tested only while breathing room air.

### Experimental procedures

#### Brachial artery hemodynamics

Brachial artery blood velocity and diameter were measured using duplex ultrasound (Terason t3200^TM^, Teratech, Burlington, MA, USA) with a 10-MHz multifrequency linear array probe. Images of the brachial artery were acquired about 5 cm proximal to the antecubital fossa. Anterograde and retrograde shear rate (SR; 4·Velocity/Diameter) was calculated from anterograde and retrograde area under the curve data that were subsequently averaged from positive and negative data points, respectively.

#### Flow-mediated dilation

Endothelium-dependent vasodilator function of the brachial artery of the experimental arm was assessed before and after each retrograde shear stress intervention according to international guidelines^38^. A rapid inflation and deflation blood pressure cuff was positioned on the forearm of the experimental arm immediately distal to the olecranon process. Baseline images of brachial artery velocity and diameter were recorded for 1 minute prior to cuff inflation. The forearm cuff was then inflated to 220 mmHg for 5 minutes. Continuous diameter and blood velocity recordings resumed 30 s prior to cuff deflation and continued 5 minutes thereafter. These procedures were repeated immediately after the intervention (i.e. 20 minutes of occlusion), and the same experienced ultrasonographer (OFB) was involved in each assessment for a given subject. The same custom-designed edge-detection and wall-tracking software as used for the Brachial Artery Blood Flow Measures was utilised for offline measurements of baseline diameter, blood flow, peak diameter and SR. Peak diameter after cuff deflation was automatically detected according to an algorithm that identified the maximum bracket of data subsequent to a moving window-smoothing function^39^. The time to peak diameter (s) was calculated from the point of cuff deflation to the maximum post-deflation diameter^39^. Diameter data are presented as absolute (FMD, milimeters) and relative (FMD%, percentage) rise of peak diameter from the preceding baseline diameter. In accordance with procedural recommendations^40^, we also measured the post-deflation area under curve for the shear rate response in order to best interpret any changes in FMD.

#### Plasma microparticle measures

Blood samples were obtained into 8 mL Cyto-chex tubes (Streck, Inc., Medimark Europe, Grenoble, France), coded and sent by express mail to the University of Maryland where all analyses were performed within 48 hours after arrival, blinded to the laboratory, as described in detail elsewhere^41,42^. Microparticle characteristics remain unchanged when samples stored at either 4 °C or at room temperature are processed in a time span of 3 weeks from time of collection^42^. The blood was centrifuged for 5 minutes at 1500 g, the supernatant was made 12.5 mM EDTA and then centrifuged at 15000 g for 30 minutes. Aliquots of the 15,000 g supernatant were stained with antibodies for MPs analysis by flow cytometry. Endothelial microparticles (EMP) were defined as those that bound annexin V and expressed CD31 (platelet-endothelial cell adhesion molecule, PECAM) but were negative for CD41 (the platelet-specific integrin alpha chain protein), and those that bound annexin V and positive for CD62E (E-selectin, a protein only expressed by endothelial cells after cytokine stimulation). Other MPs were characterized as those binding annexin V and expressing CD41, CD142 (tissue factor), CD66b (Carcinoembryonic antigen-related cell adhesion molecule 8, a protein expressed exclusively by polymorphonuclear leukocytes, PMN), and von Willebrand factor (vWF). Flow cytometry analysis of MPs was performed with an 8-color, triple laser MACSQuant (Miltenyi Biotec Corp., Auburn, CA) using the manufacturers’ acquisition software. All reagents and solutions used for MPs analysis were sterile and filtered (0.2 μm filter). MPs were stained with annexin V and analyzed as previously described, including micro-beads with diameters of 0.3 μm (Sigma, Inc.), 1.0 μm, and 3.0 μm (Spherotech, Inc., Lake Forest, IL) to assess the size of particles^41,42^. Surface markers were evaluated with use of the “Fluorescence-Minus-One Control Test”. We define MPs as annexin V-positive particles with diameters up to 1 µm as previously described^41,42^. In previous analyses the variability between repeated measures in the same samples was below 5%. We have also run a control sample to assure we use the correct gating protocol and a suspension of polystyrene fluorescent beads to be sure the particle counts are correct.

#### Statistical analysis

All data are reported as mean ± SD with statistical significance set at P < 0.05. For shear rate analysis, we used a 3-way ANOVA to determine the difference between COPD patients and control subjects, room air and 100% oxygen condition and the impact of cuff inflation on shear rate (pre intervention, 10^th^ and 20^th^ minute of intervention).

The impact of elevated retrograde shear stress and oxygen normalization on FMD (experimental arm) in COPD subjects was assessed using a 2-way repeated measures ANOVA (factors: room air vs. oxygen breathing and pre vs. post retrograde shear). The impact of elevated retrograde shear stress and oxygen normalization on MPs (experimental arm), and MPs (control arm) were assessed using a Friedman repeated measures ANOVA on ranks. Post-hoc t–tests were performed if a main effect was found using Tukey’s HSD correction for multiple comparisons. The relationship between baseline FMD and baseline retrograde shear in patients and controls was tested with Pearson’s correlation test.

The impact of retrograde shear stress on FMD in control subjects breathing room air was assessed using a two-tailed paired t-test. The impact of retrograde shear stress on MP levels in controls was analysed using a 2-way mixed design ANOVA (between subject factor: control vs. experimental arm; within subject factor: pre vs. post retrograde shear). To account for stimulus differences, FMD was compared using a 2-way ANCOVA (pre- and post-intervention during room air and O_2_ administration) in COPD patients with logarithmically-transformed baseline diameter and shear rate area under the curve (SRAUC) as covariates. Post-hoc t–tests were performed if a main or interaction effect was found using a Bonferroni correction for multiple comparisons. The effects of mean arterial pressure (MAP), oxygen saturation (SaO_2_) and the ratio of forced expiratory volume in the first second and forced vital capacity (FEV_1_/FVC ratio) on baseline FMD, FMD response to oscillatory shear and low flow oxygen supplementation were tested as covariates. Cohen’s *d* was used to compute the effect size of the difference between the two population means.
